# Disulfidptosis and its emerging relevance in cancer and immunity

**DOI:** 10.70401/fos.2025.0004

**Published:** 2025-11-18

**Authors:** Qidong Li, Shengrong Wu, Li Zhuang, Boyi Gan

**Affiliations:** 1 Department of Experimental Radiation Oncology, The University of Texas MD Anderson Cancer Center, Houston, TX 77030, USA.; 2 The University of Texas MD Anderson UTHealth Graduate School of Biomedical Sciences, Houston, TX 77030, USA.

**Keywords:** Disulfidptosis, SLC7A11, ferroptosis, cancer therapy, immunity, cell death biomarker

## Abstract

Disulfidptosis is a recently identified form of regulated cell death (RCD) triggered by disulfide stress when cystine uptake via solute carrier family 7 member 1 (SLC7A11) overwhelms the cell’s reducing capacity. Unlike apoptosis or other “cell suicide” pathways, disulfidptosis likely represents a “cell sabotage” mechanism, defined by aberrant disulfide bonding and catastrophic actin cytoskeleton collapse. In this Perspective, we examine the paradoxical role of SLC7A11 as both a ferroptosis protector and a disulfidptosis trigger, and the mechanistic hallmarks of disulfidptosis. We highlight emerging therapeutic strategies to target disulfidptosis in cancer, including glucose transporter inhibition, redox-targeting agents, and nanomaterial-based approaches, and consider its dual role in immunity, where it may suppress T cell function yet act as a form of immunogenic cell death. Together, these insights position disulfidptosis as both a conceptual advance in RCD biology and a promising target for cancer therapy that warrants further mechanistic and translational exploration.

## 1. Introduction

Regulated cell death (RCD) is central to organismal development, tissue homeostasis, and protection against stress and infection^[1]^. For many years, apoptosis has served as the dominant model of RCD, illustrating how cells can undergo a genetically programmed route to self-destruction in response to diverse cues. More recently, however, this view has broadened considerably. Over the past two decades, additional forms of RCD, including necroptosis, pyroptosis, ferroptosis, and cuproptosis, have been identified, each defined by distinct molecular triggers and execution mechanisms linked to cellular metabolism, immune pathways, or environmental stress^[2]^. These advances have expanded our understanding of how cells die, revealed unexpected links between cell death and disease pathophysiology, and opened new avenues for therapeutic targeting in cancer, neurodegeneration, and other diseases.

Within this evolving framework, disulfidptosis has recently emerged as a distinct form of RCD. First described in 2023 by our group, disulfidptosis refers to a form of RCD triggered by disulfide stress—an imbalance in thiol-disulfide homeostasis that arises under oxidative conditions when cystine uptake exceeds the cell’s reducing capacity^[3,4]^. At the conceptual level, we propose that disulfidptosis, together with ferroptosis and cuproptosis, belongs to a broader class of “cell sabotage” pathways^[5]^. This type of RCD is induced when essential metabolic processes become disrupted, leading to lethal cellular self-destruction as a byproduct of core metabolic imbalances^[5,6]^. This contrasts with “cell suicide” pathways such as apoptosis, necroptosis, and pyroptosis, where cell death is orchestrated by genetically encoded signaling cascades and executioner proteins (e.g., caspases, mixed lineage kinase domain-like protein (MLKL), and Gasdermins) in a programmatic and orderly manner^[5]^. Stated differently, “cell suicide” reflects an internally regulated decision to die, whereas “cell sabotage” reflects catastrophic collapse of homeostatic systems that cells rely on to survive. At the mechanistic level, these sabotage pathways are unified by metabolic imbalance but differ in their metabolic triggers^[7]^: ferroptosis is driven by iron-dependent lipid peroxidation^[8]^, cuproptosis by copper-induced aggregation of lipoylated mitochondrial proteins and depletion of iron-sulfur cluster proteins^[9]^, and disulfidptosis by catastrophic disruption of the actin cytoskeleton caused by aberrant disulfide bond formation^[10]^.

Over the past two years since its discovery, disulfidptosis has attracted increasing attention in the research community, both for its mechanistic novelty and its potential therapeutic relevance in cancer and immunity. This Perspective provides a detailed examination of the disulfidptosis field at its current stage. We first outline the paradoxical role of the cystine/glutamate antiporter solute carrier family 7 member 11 (SLC7A11, also known as xCT) in regulating ferroptosis and disulfidptosis, followed by a discussion of the mechanistic hallmarks of disulfidptosis. We then consider the emerging translational implications of targeting disulfidptosis in cancer therapy and discuss a surprising recent finding revealing disulfidptosis as a driver of immune dysfunction, particularly in exhausted CD8^+^ T cells. Finally, we discuss challenges in biomarker development, unresolved questions, and directions for future research. Together, these discussions illustrate how disulfidptosis both enriches the broad conceptual framework of RCD and opens new avenues for understanding metabolic vulnerabilities in cancer and immunity.

## 2. SLC7A11 Paradox: Ferroptosis Suppressor and Disulfidptosis Trigger

**Figure 1.**
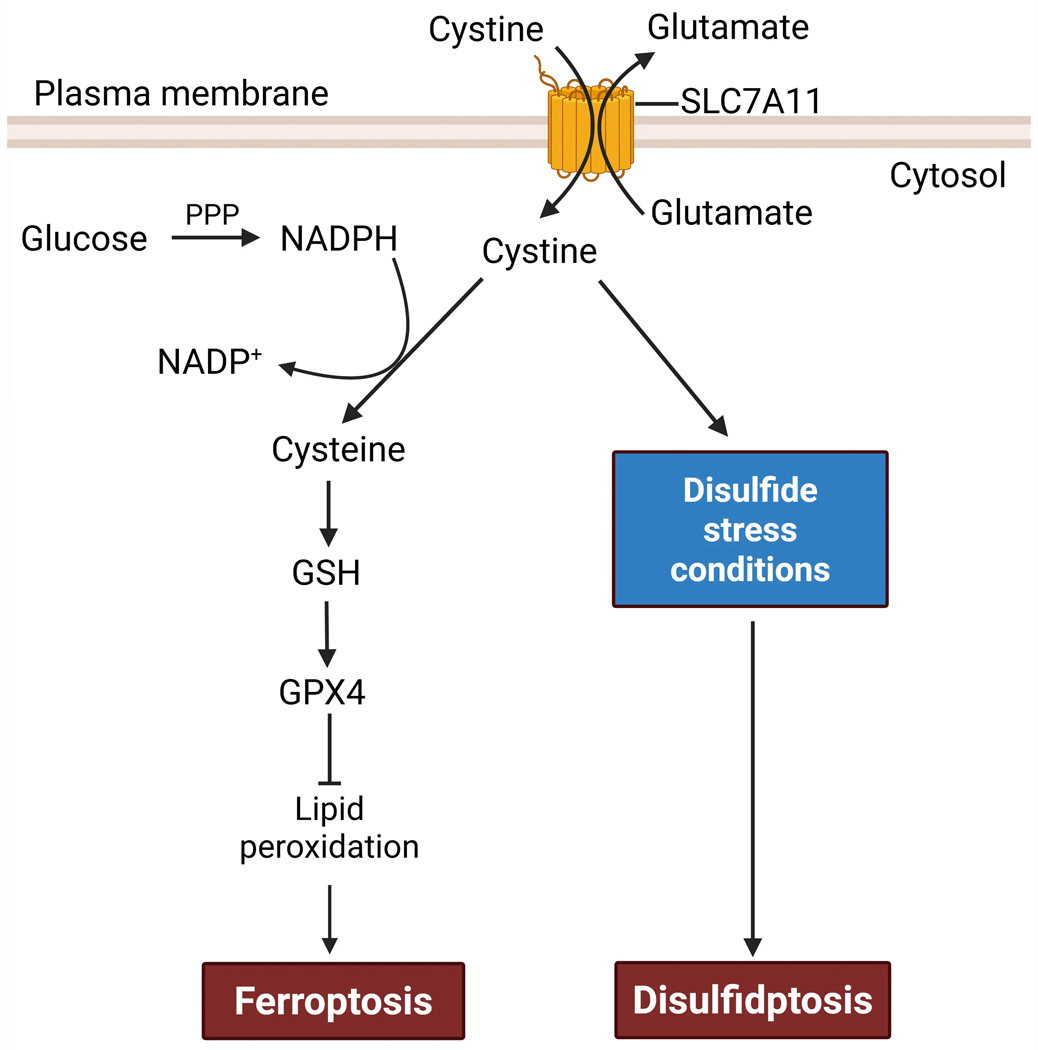
A dual role of SLC7A11 in regulating ferroptosis versus disulfidptosis. SLC7A11 acts as an antiporter that imports extracellular cystine and exports intracellular glutamate. Once inside cells, cystine is reduced to cysteine, which supports GSH- and GPX4-dependent suppression of lipid peroxidation, thereby inhibiting ferroptosis. Therefore, SLC7A11 acts as a negative regulator of ferroptosis. Cystine reduction to cysteine consumes the reducing equivalent NADPH, which is mainly generated via glucose-fueled PPP. Under disulfide stress conditions, such as glucose deprivation or PPP inhibition in SLC7A11-high cells, cystine reduction is impaired, leading to aberrant accumulation of cystine and induction of disulfidptosis. Consequently, SLC7A11 has a promoting role in disulfidptosis. Created in BioRender. SLC7A11: solute carrier family 7 member 1; GSH: synthesis of glutathione; GPX4: glutathione peroxidase 4; NADPH: reduced nicotinamide adenine dinucleotide phosphate; PPP: pentose phosphate pathway.

At the center of disulfidptosis lies a paradoxical feature of SLC7A11, the light chain subunit of the antiporter system x_c_-, which imports extracellular cystine in exchange for intracellular glutamate^[11,12]^ (Figure 1). In most contexts, high SLC7A11 activity is cytoprotective: once imported into the cytosol via SLC7A11, cystine (the oxidized dimer of cysteines linked by a disulfide bond) is reduced to cysteine, which then provides the key precursor for the synthesis of glutathione (GSH), the cell’s major antioxidant^[12]^. GSH in turn serves as an essential cofactor for glutathione peroxidase 4 (GPX4), which detoxifies lipid hydroperoxides by converting them into lipid alcohols^[13]^ (Figure 1). In this way, SLC7A11 protects cells from ferroptosis, a form of RCD triggered by lipid peroxidation when antioxidant defenses become defective (for example, upon inhibition of SLC7A11 or GPX4)^[12,14]^. Indeed, upregulation of SLC7A11 is a common adaptive response to oxidative stress and a feature of many cancers, particularly those with *kelch-like ECH-associated protein 1* (*KEAP1*) mutations or constitutive nuclear factor erythroid 2-related factor 2 (NRF2) activation, which upregulates the expression of antioxidant genes, including *SLC7A11*^[12,15]^.

**Figure 2.**
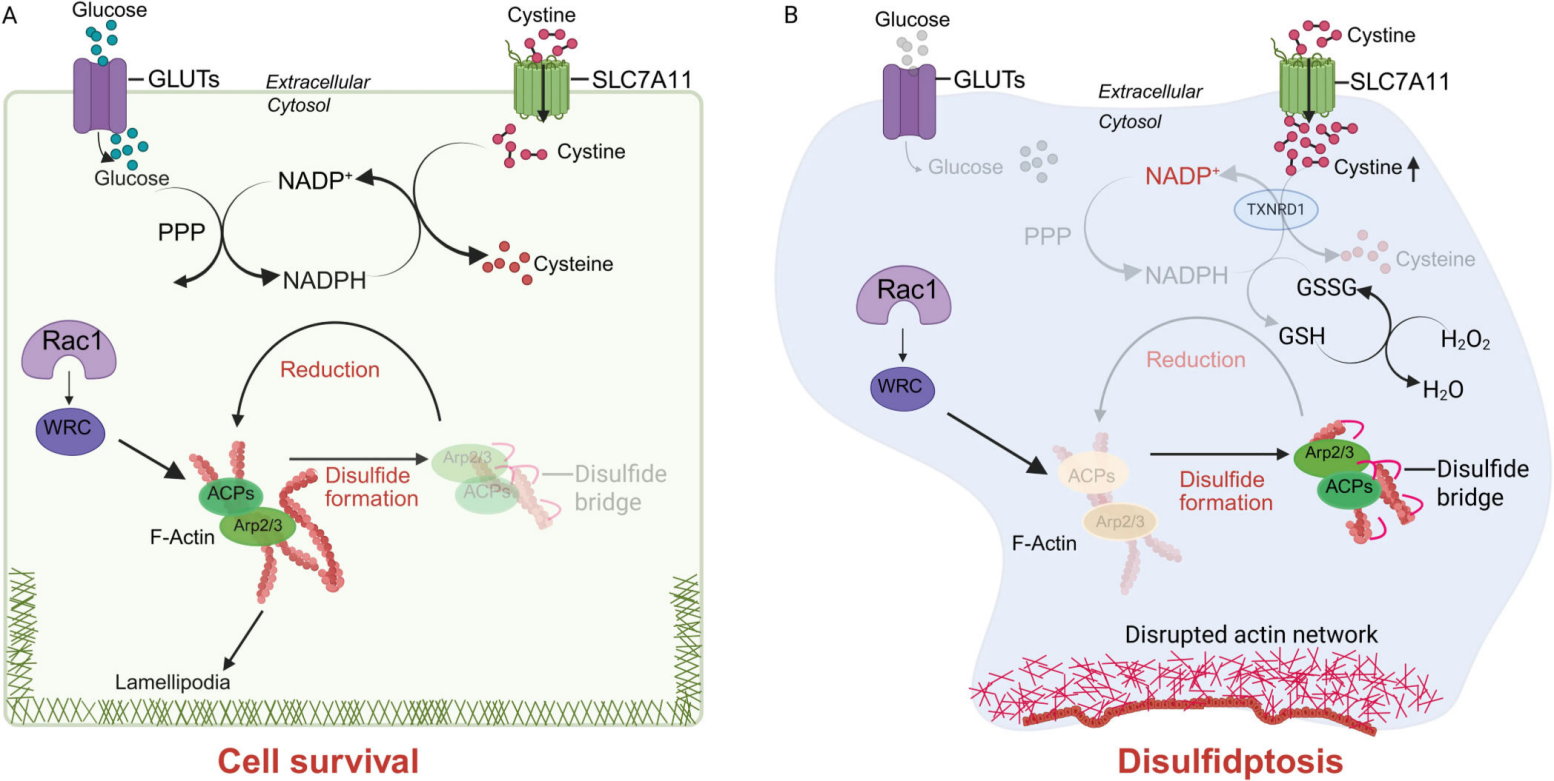
The disulfidptosis pathway. (A) Under glucose-replete conditions, the PPP continuously produces NADPH, which provides the reducing power required for the conversion of SLC7A11-imported cystine into cysteine. This reduction process prevents aberrant disulfide bond accumulation in ACPs, thereby maintaining cytoskeletal stability and ensuring cell survival; (B) In contrast, glucose deprivation disrupts PPP activity and severely limits NADPH availability in SLC7A11-high cells. Sustained cystine import under these conditions leads to excessive intracellular accumulation of cystine and other disulfide-containing metabolites, which promote abnormal disulfide bond formation within ACPs. The resulting collapse of the actin network initiates disulfidptosis. Inhibition of TXNRD1, which mediates cystine reduction to cysteine or H_2_O_2_ treatment has also been shown to induce disulfidptosis in SLC7A11-high cells. Furthermore, the Rac1-WRC-Arp2/3 axis drives branched actin polymerization and lamellipodia formation, which are thought to provide a structural platform that facilitates disulfide bond formation in ACPs, thereby amplifying disulfidptosis. In the figure, disulfide bonds are illustrated as small red arcs. Created in BioRender. PPP: pentose phosphate pathway; NADPH: reduced nicotinamide adenine dinucleotide phosphate; SLC7A11: solute carrier family 7 member 1; ACPs: actin cytoskeleton proteins; TXNRD1: Thioredoxin reductase 1.

Surprisingly, in 2017 several groups, including ours, reported that SLC7A11 overexpression potently promotes glucose starvation–induced cell death, whereas its deletion markedly suppresses it in many cancer cell types, especially those with high SLC7A11 expression^[16–18]^. Further research uncovered that while SLC7A11-mediated cystine uptake protects cells against ferroptosis, it also creates a metabolic liability: imported cystine is poorly soluble and chemically reactive, and thus must be rapidly reduced to cysteine, a process requiring the reducing equivalent reduced nicotinamide adenine dinucleotide phosphate (NADPH)^[19,20]^. Under glucose-replete conditions, the pentose phosphate pathway (PPP) provides sufficient NADPH to support cystine reduction (Figure 2A). However, under metabolic stress, such as glucose starvation or strong oxidative insults (e.g., H_2_O_2_ treatment), NADPH becomes limiting, leaving large amounts of imported cystine unreduced in SLC7A11-high cells; consequently, disulfide species accumulate, disrupting thiol homeostasis and driving disulfide stress^[19–21]^ (Figure 2B). This stress causes aberrant covalent cross-linking of actin and actin-binding proteins, leading to cytoskeleton collapse and ultimately disulfidptosis^[3,22]^ (Figure 2B).

This dual role of SLC7A11 reinforces a broader principle in cell biology that cell fate is often context-dependent; a pathway that is protective in one cellular condition may become detrimental in another. From a therapeutic perspective, such paradoxes can be explored in cancer treatment: tumors that upregulate SLC7A11 for ferroptosis resistance may simultaneously render themselves vulnerable to disulfidptosis. Supporting this idea, recent work demonstrated that glucose transporter 1 (GLUT1) inhibition selectively induces disulfidptosis and suppresses the growth of SLC7A11-high, *KEAP1*-mutant tumors^[3,19,23]^. As another example, the tumor suppressor BRCA1-associated protein 1 (BAP1) suppresses *SLC7A11* expression, and *BAP1*-mutant cancer cells, due to their elevated expression levels of SLC7A11, are resistant to ferroptosis but are susceptible to glucose starvation–induced disulfidptosis^[24,25]^, suggesting a therapeutic strategy for targeting *BAP1*-mutant tumors. Recent findings also showed that valproic acid, a clinically used histone deacetylase inhibitor, promotes ferroptosis while suppressing disulfidptosis in hepatocellular carcinoma^[26]^, highlighting how the same metabolic axis can promote or suppress different RCD modalities depending on cellular and pharmacological context. Thus, the SLC7A11 paradox exemplifies how metabolic adaptations that initially promote cell survival can create unexpected vulnerabilities that may be explored for cancer therapy.

## 3. Mechanistic Hallmarks: Cytoskeleton Collapse Under Disulfide Stress

A key feature of disulfidptosis is the accompanying cytoskeleton catastrophe^[3,22]^. Unlike apoptosis, which involves caspase-mediated cleavage of substrates, or ferroptosis, which culminates in lipid bilayer damage caused by excessive lipid peroxidation, disulfidptosis is induced, at least in part, by aberrant disulfide bond formation in cytoskeletal proteins. Importantly, disulfidptosis is not blocked by inhibitors of apoptosis, necroptosis, or ferroptosis, further confirming its mechanistic distinctness^[3]^. It is also induced rapidly, often within hours of metabolic perturbation (such as upon glucose starvation in SLC7A11-high cancer cells)^[3]^, suggesting that it does not rely on transcriptional reprogramming but might result directly from redox imbalance at the protein level. Consistently, proteomic analyses have revealed prominent disulfide bonding in cytoskeletal proteins such as filamin A and B, myosin-9, Drebrin, and actin itself, which likely contribute to a rapid collapse of the actin filament network, loss of cellular integrity, and disulfidptotic cell death^[3]^.

Why is the cytoskeleton particularly susceptible to disulfide stress? One potential explanation is the high density of reactive cysteine residues within actin and actin-associated proteins. Under normal conditions, these residues are tightly regulated by thiol–disulfide exchange systems, ensuring structural flexibility while preventing irreversible cross-linking. However, in a disulfide-stressed environment (such as in glucose-starved SLC7A11-high cells), these safeguards are overwhelmed, and disulfide bonds accumulate within these proteins. The cytoskeleton, normally a hub of adaptability, becomes rigid and dysfunctional, leading to cellular demise.

Beyond cancer, emerging evidence suggests that disulfidptosis may also occur in physiological or nutritional stress contexts. For example, suppression of selenoprotein T in selenium-deficient skeletal muscle was recently shown to trigger disulfidptosis through mitochondrial reactive oxygen species (ROS)–NADPH dysregulation, thereby linking dietary micronutrient imbalance to thiol–disulfide collapse^[27]^. This finding extends the relevance of disulfidptosis beyond cancer biology and raises the possibility that it could contribute to tissue injury in settings such as nutrient deficiency or metabolic disease.

The Rac–WAVE regulatory complex (WRC) signaling axis, which regulates Arp2/3-mediated branched actin polymerization, appears to promote this cell death pathway^[3]^ (Figure 2B). Overexpression of constitutively active Rac was found to enhance disulfidptosis, whereas deletion of protein components in the WRC partially protects cells from disulfidptosis^[3]^. It is possible that, by generating dense networks of branched actin filaments, the Rac–WRC may increase the local concentration of cysteine-rich structures susceptible to disulfide bonding.

Nevertheless, several mechanistic questions remain unresolved. For example, are there existing upstream sensors that detect disulfide accumulation and actively promote execution, or is disulfidptosis purely a passive collapse? Are there “executioner” enzymes that catalyze aberrant disulfide bond formation, similar to caspases in apoptosis? Is there an additional mechanism that operates independent of or in parallel to the actin cytoskeleton and mediates disulfidptosis? Identifying these molecular players will be key to understanding whether disulfidptosis is an actively regulated process or simply a metabolic accident in cells.

## 4. Therapeutic Implications: Exploring Disulfidptosis in Cancer

The discovery of disulfidptosis has immediate implications for cancer therapy. Many tumors, such as those with *BAP1* or *KEAP1* mutations or NRF2 hyperactivation, exhibit high SLC7A11 expression^[12]^. While this adaptation protects these tumors from ferroptosis, it renders them uniquely dependent on a continuous NADPH supply for cystine reduction and vulnerable to disulfide stress. This suggests a therapeutic strategy that perturbes NADPH supply or increases cystine load might selectively drive SLC7A11-high cancer cells into disulfidptosis. Below, we summarize several strategies that have been tested in preclinical models in recent studies (Table 1).

### Glucose transport inhibition.

**Figure 3.**
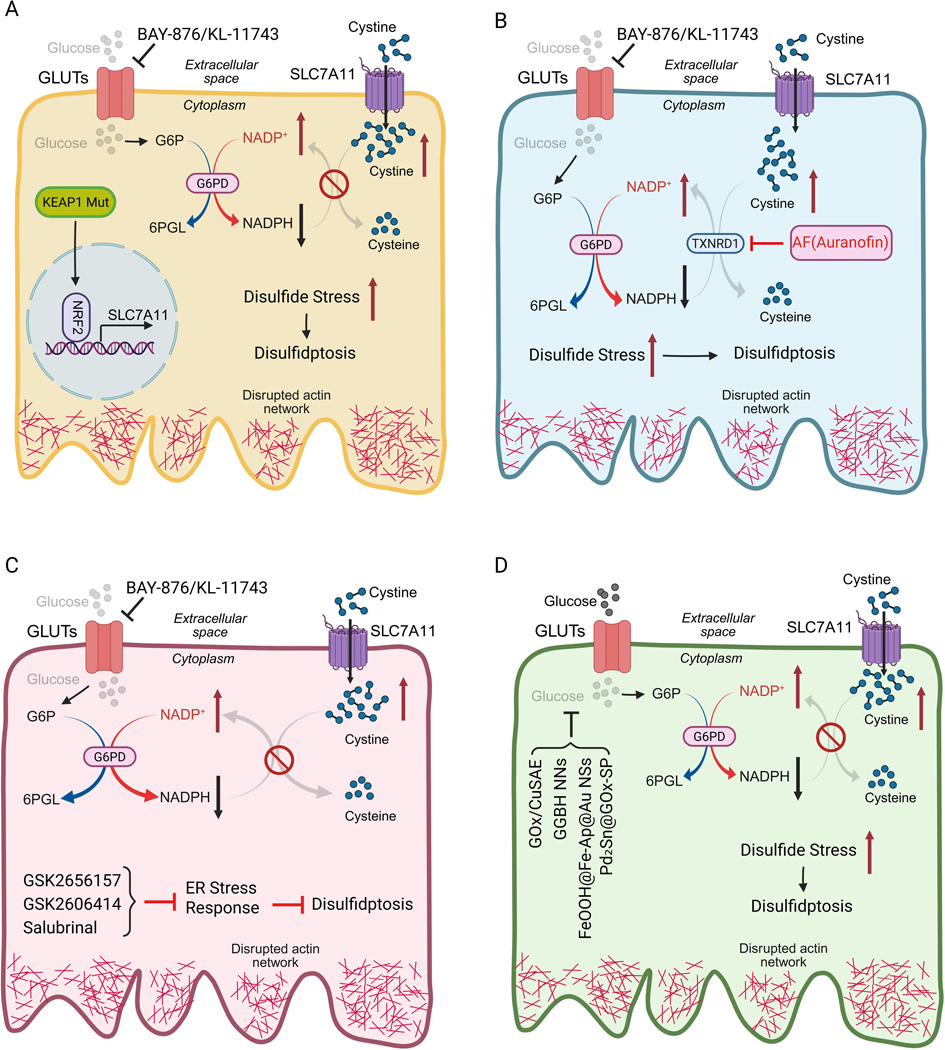
Disulfidptosis-inducing strategies in cancer therapy. (A) Glucose transport inhibition: pharmacological blockade of GLUTs using BAY-876 or KL-11743 suppresses glucose uptake and PPP flux, thereby inducing robust disulfidptosis and inhibiting tumor growth in *KEAP1*-mutant lung cancers (which exhibit constitutively active NRF2 and aberrant expression of SLC7A11); (B) Redox system inhibition: TXNRD1 converts cystine to cysteine. Inhibition of TXNRD1 by Auranofin is sufficient to trigger disulfidptosis in cells with high SLC7A11 expression. When combined with glucose starvation, this approach also sensitizes cells with intermediate SLC7A11 levels to disulfidptotic cell death; (C) Other combination strategies: co-treatment with BAY-876 or KL-11743 and agents that modulate ER stress or elevate oxidative stress effectively promotes disulfidptosis and suppresses tumor growth; (D) Nanomaterial-based strategies: nanomaterial-based therapeutics targeting glucose metabolism impair PPP-derived NADPH generation, thereby lowering reducing capacity and driving disulfidptosis. Created in BioRender. GLUTs: glucose transporters; PPP: pentose phosphate pathway; *KEAP1*: *kelch-like ECH-associated protein 1*; SLC7A11: solute carrier family 7 member 1; TXNRD1: Thioredoxin reductase 1; ER: endoplasmic reticulum; NADPH: reduced nicotinamide adenine dinucleotide phosphate.

Blocking GLUTs with compounds such as BAY-876 or KL-11743 inhibits glucose uptake and PPP activity. In *KEAP1*-mutant lung cancers, this approach induced pronounced disulfidptosis and suppression of tumor growth^[23]^ (Figure 3A). Consistent with this, recent studies showed that the cancer susceptibility candidate 8 (lncRNA *CASC8*) promotes PPP flux via the c-Myc–GLUT1 axis, thereby suppressing disulfidptosis in pancreatic ductal adenocarcinoma^[28]^, and pan-cancer analyses revealed that glucose deprivation induces disulfidptosis through the SLC7A11–INF2 axis^[29]^.

### Redox system inhibition.

Thioredoxin reductase (TXNRD1) has been proposed to reduce cystine to cysteine; consequently, TXNRD1 inhibition by Auranofin treatment, which would block the conversion of cystine to cysteine and exacerbate disulfide accumulation, induces disulfidptosis in SLC7A11-high contexts^[30]^, or, when combined with glucose starvation, this triggers disulfidptosis even in cells with moderate SLC7A11 expression^[31]^ (Figure 3B). Relatedly, Gaudichaudione H, a natural small-molecule compound derived from Garcinia oligantha Merr. (Clusiaceae), was shown to enhance disulfidptosis sensitivity in hepatocellular carcinoma by modulating the NRF2-SLC7A11 signaling pathway^[32]^. These studies support the feasibility of combining redox-targeting agents with metabolic interventions.

### Combination strategies.

Combining GLUT inhibitors with endoplasmic reticulum (ER) stress modulators or oxidative stress inducers has been shown to enhance anti-tumor efficacy in preclinical models^[33]^ (Figure 3C), suggesting that rational combinations may produce durable responses while minimizing resistance.

### Nanomaterial-based strategies.

Multiple recent studies have explored nanotechnology approaches to trigger or amplify disulfidptosis in tumors (Figure 3D). A copper-based nanoinducer with multiple enzyme-mimicking activities was shown to synergize disulfidptosis with pyroptosis for tumor immunotherapy^[34]^. In another study, a single-atom nanozyme that perturbs energy supply and reducing power effectively triggers tumor disulfidptosis^[35]^. More recently, a multifunctional nanoplatform—FeOOH nanoshuttles coloaded with Fe-apigenin complexes and Au nanodots (FeOOH@Fe-Ap@Au NSs), was developed to simultaneously induce disulfidptosis and ferroptosis^[36]^. Another recent study reported that Pd₂Sn intermetallic nanorods, modified with glucose oxidase and phospholipids (Pd₂Sn@GOx-SP), can simultaneously promote pyroptosis and disulfidptosis, thereby enhancing anti-tumor immunity^[37]^. This dual-targeting strategy highlights how nanomaterials can be engineered to simultaneously manipulate multiple RCD pathways, offering a new approach for cancer therapy. Furthermore, unlike traditional pharmacological approaches, nanomaterial-based systems can be engineered to integrate multiple catalytic or delivery functions, which provide unique opportunities to precisely manipulate redox balance and cell death pathways in tumors.

These therapeutic opportunities are particularly attractive because they target cancers often considered difficult to treat. For example, *BAP1*-mutant renal cancers or *KEAP1*-mutant non-small cell lung cancers respond poorly to standard chemotherapy, radiation, and immunotherapy^[38,39]^. By exploiting their metabolic liability through disulfidptosis induction, new treatment strategies could be developed for these otherwise lethal diseases.

Disulfidptosis induction offers a unique therapeutic entry point that targets the redox and metabolic vulnerabilities of tumor cells. Conceptually, it may synergize with radiotherapy or chemotherapeutic agents that elevate ROS, thereby amplifying disulfide stress and lowering the redox threshold for cell death. Likewise, immune checkpoint blockade could be enhanced by disulfidptosis-induced release of oxidative damage-associated signals, potentially boosting dendritic-cell activation and cytotoxic T-cell responses. However, as discussed in a later section, excessive disulfide stress might also impair immune cell viability or function; therefore, a careful balance between tumor-selective induction and immune preservation will be critical in designing such combinations. Another promising avenue is to pair disulfidptosis inducers with ferroptosis or cuproptosis inducers, exploiting convergent oxidative stress pathways to overcome single-pathway resistance.

Since disulfidptosis was discovered only recently, to our knowledge, no ongoing clinical trials are explicitly designed to evaluate disulfidptosis induction as a therapeutic mechanism. Moving forward, several challenges need to be addressed in translating these preclinical findings into the clinic. First, although SLC7A11-high tumors are expected to be more sensitive to disulfidptosis than normal tissues (which generally express low levels of SLC7A11), the risk of systemic toxicity cannot be ignored, as disulfide stress may still impact healthy tissues. In addition, tumors may evolve resistance to disulfidptosis-inducing therapy, for example by downregulating SLC7A11 or activating alternative NADPH-generating pathways such as malic enzyme 1 or isocitrate dehydrogenase 1^[40]^. Furthermore, defining predictive biomarkers, as discussed later, will be essential for identifying patients most likely to benefit from disulfidptosis-inducing treatments. Finally, as discussed in the next section, the immunological consequences of disulfidptosis induction still remain largely unknown and might complicate how we target this form of cell death in cancer.

## 5. Disulfidptosis and Immunity: A Double-Edged Sword

Another important recent advance in disulfidptosis research is the recognition that disulfidptosis is not limited to cancer cells but also occurs in immune cells, particularly exhausted CD8^+^ T lymphocytes within the tumor microenvironment. While earlier studies have mainly used cancer cells cultured in glucose-free medium to study disulfidptosis, a recent work demonstrated that exhausted CD8^+^ T cells can undergo disulfidptosis within their native microenvironment even under physiological glucose conditions^[41]^. Therefore, disulfidptosis acts not only as a vulnerability in tumors but also as a potential mechanism of immune suppression within the tumor microenvironment^[42]^.

**Figure 4.**
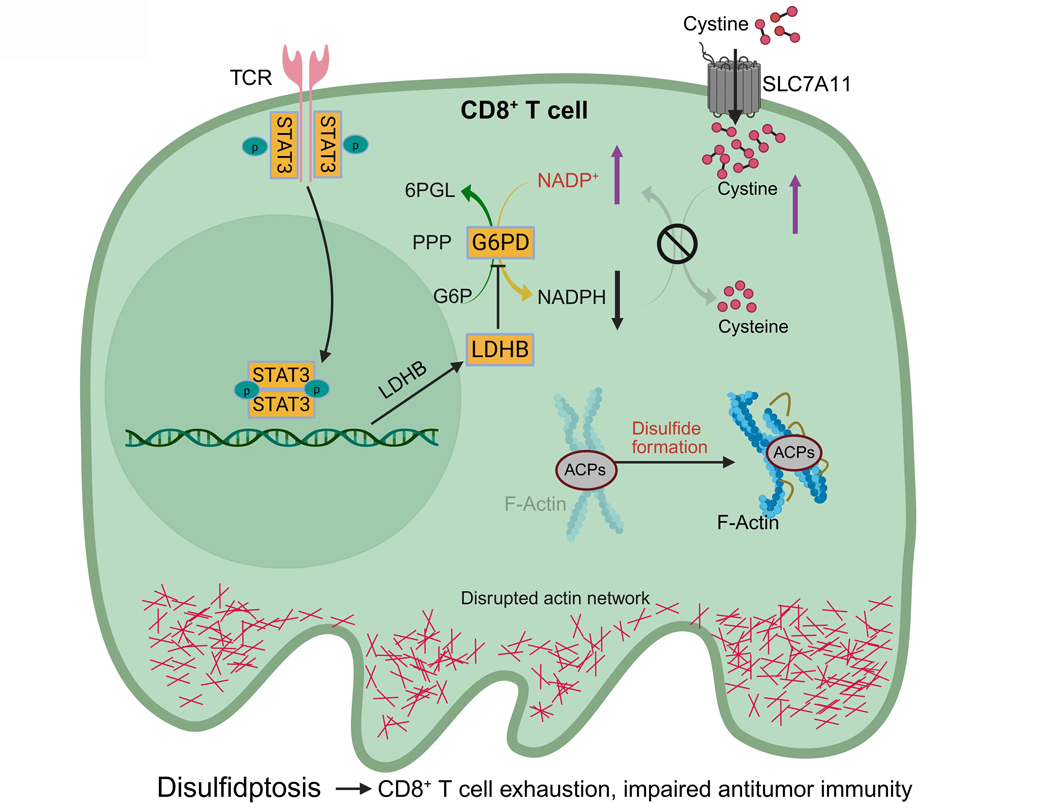
Interplay between disulfidptosis and antitumor immunity. SLC7A11 imports cystine into CD8^+^ T cells, where NADPH generated by the PPP reduces it to cysteine, preventing disulfide stress and preserving cytoskeletal integrity. During CD8^+^ T cell exhaustion, chronic T cell receptor signaling activates STAT3, which induces LDHB expression. LDHB inhibits G6PD, the rate-limiting enzyme in the PPP that converts G6P to 6-phosphogluconolactone while generating NADPH, thereby suppressing PPP-derived NADPH. When NADPH is depleted, cystine accumulates, causing aberrant disulfide bonding in ACPs, actin network collapse, and disulfidptosis in CD8^+^ T cells. This accelerates T cell exhaustion and weakens antitumor immunity. Created in BioRender. SLC7A11: solute carrier family 7 member 1; NADPH: reduced nicotinamide adenine dinucleotide phosphate; STAT3: signal transducer and activator of transcription 3; LDHB: lactate dehydrogenase B; G6PD: glucose-6-phosphate dehydrogenase; G6P: glucose-6-phosphate; PPP: pentose phosphate pathway; ACPs: actin cytoskeleton proteins.

Mechanistically, the study identified a signal transducer and activator of transcription 3 (STAT3)–lactate dehydrogenase B (LDHB)–glucose-6-phosphate dehydrogenase (G6PD) signaling axis that mediates disulfidptosis in exhausted CD8^+^ T cells^[41]^ (Figure 4). STAT3, a transcription factor involved in T cell exhaustion, upregulates LDHB, which then binds to and inhibits G6PD, the rate-limiting enzyme of the PPP. This suppresses NADPH production, impairing cystine reduction and predisposing T cells to disulfide stress.

Consequently, cystine accumulation triggers aberrant disulfide bonding in actin, collapsing the cytoskeleton and inducing T cell death via disulfidptosis or dysfunction (Figure 4). As a result, disulfidptosis contributes to T cell exhaustion, a state in which CD8^+^ T cells lose their cytotoxic function and fail to control tumor growth. In support of this, heterozygous deletion of *Slc7a11* in T cells mitigated disulfidptosis and improved antitumor immunity, suggesting that downregulating SLC7A11-mediated cystine uptake could preserve T cell function^[41]^.

These findings complicate therapeutic applications of disulfidptosis. While inducing this form of cell death in cancer cells may be beneficial, its immunosuppressive effect in T cells could undermine immune responses. This role of disulfidptosis poses a potential challenge for translational development: how can we selectively induce tumor disulfidptosis while sparing immune cells? Potential solutions include tumor-specific delivery systems, biomarker-based patient selection, or combination therapies that protect T cells while promoting tumor cell death.

Notably, recent studies also suggest that disulfidptosis may be a form of immunogenic cell death (ICD), releasing signals that activate dendritic cells and prime antitumor immunity^[31]^. This would align disulfidptosis with ferroptosis, which can also be immunogenic under certain contexts^[15]^. Whether disulfidptosis is ultimately immunosuppressive or immunostimulatory (or both, depending on the cell type and context) remains a critical question for future work.

## 6. Biomarkers and Detection: The Missing Toolkit

For any novel cell death pathway to advance toward clinical translation, robust biomarkers are essential. For example, cleaved caspase-3 has long served as the gold standard for measuring apoptosis in both basic research and clinical settings. However, disulfidptosis detection currently lacks simple, robust, and reliable markers. At present, detection of disulfidptosis relies on a combination of several techniques and experimental approaches:

- **Non-reducing Western blotting** to detect aberrant disulfide bonding in selected cytoskeletal proteins.
- **Metabolomic profiling** to measure accumulation of cystine and other disulfide molecules and indicators of redox imbalance (e.g., increased NADP^+^/NADPH ratio).
- **Fluorescent staining of F-actin** (e.g., phalloidin staining) to demonstrate the characteristic disruption of actin cytoskeleton structure.
- **Rescue experiments in cell and animal models** to show that the studied cell death or disulfidptosis-associated phenotypes can be suppressed by *SLC7A11* deletion or inhibition, or by reducing agents such as dithiothreitol, β-mercaptoethanol, or tris(2-carboxyethyl)phosphine, but not by inhibitors of other cell death pathways, such as apoptosis or ferroptosis.

Interpretation is further complicated by the fact that disulfidptosis is often studied under glucose starvation, a metabolic stress condition that can also trigger apoptosis or other forms of RCD in SLC7A11-low cells. This overlap raises the possibility that some reported cases of “disulfidptosis” in the literature may, in fact, reflect other cell death modalities. Therefore, to ensure rigorous and accurate detection, it is critical to integrate multiple approaches as outlined above rather than relying on a single assay.

Furthermore, while these tools are informative for research, they are not readily adaptable for clinical application. Oxidative stress from unrelated processes could also produce overlapping signatures, raising specificity concerns. To enable clinical translation, the field will need biomarkers that are both selective and clinically compatible. For example, positron emission tomography (PET) imaging with [^18^F]FSPG^[43]^, which measures SLC7A11-mediated cystine uptake, could serve as a noninvasive surrogate marker of disulfidptosis sensitivity in tumors. In parallel, redox-sensitive fluorescent or chemiluminescent probes and mass spectrometry-based disulfide proteomics could enable direct detection of aberrant disulfide bonds or actin crosslinking. Similarly, immunohistochemistry-based detection of disulfide-modified cytoskeleton proteins (or additional protein markers) in patient samples could provide pathologists with practical tools. The eventual integration of these molecular, imaging, and histologic approaches will be key to establishing a standardized biomarker framework for disulfidptosis research and clinical translation.

## 7. Conclusion and Future Directions

Disulfidptosis represents an exciting addition to the expanding repertoire of RCD mechanisms. Defined by redox-driven cytoskeletal collapse under disulfide stress, it highlights how imbalances between nutrient uptake and cellular reducing capacity can fatally disrupt cell integrity. Its paradoxical link to SLC7A11-mediated ferroptosis protection underscores a recurring theme in cancer cell biology: adaptive mechanisms that enhance survival in one context may generate lethal liabilities in another. Its role in immunity reveals both challenges and opportunities for cancer therapy, as disulfidptosis may simultaneously suppress T cell function and provide a novel form of ICD.

As with any newly discovered cell death pathway, disulfidptosis raises more questions than it answers. Some of them are highlighted below:

1. **Execution mechanisms**: A key question is whether disulfidptosis represents an actively regulated process or merely a passive biochemical collapse triggered by redox imbalance. Several observations suggest that disulfidptosis involves regulated signaling components. Cytoskeletal disulfide crosslinking preferentially affects actin and actin-regulatory proteins such as filamin and myosin, implying substrate selectivity rather than random oxidation. Moreover, the dependence of disulfidptosis on SLC7A11-mediated cystine uptake and NADPH availability further indicates metabolically gated control. However, no dedicated executioner (analogous to caspases or MLKL) has yet been identified. Thus, current evidence supports the view that disulfidptosis represents a regulated form of metabolic cell death with defined upstream triggers and structural effectors, though its precise molecular sensors and downstream mediators remain to be elucidated.
2. **Crosstalk with other RCD pathways:** Although disulfidptosis is conceptually distinct from classical “cell suicide” programs such as apoptosis, necroptosis, and pyroptosis, potential molecular intersections among these pathways remain an important but largely unexplored question. Current data indicate that SLC7A11 serves as a key regulatory node, promoting disulfidptosis under NADPH-depleted conditions while suppressing ferroptosis by maintaining glutathione synthesis. Whether disulfidptotic stress can secondarily activate apoptotic or pyroptotic cascades, or whether shared redox or metabolic signals (such as ROS accumulation, thiol oxidation, or NADPH imbalance) mediate crosstalk among these RCD pathways, remains unknown. Investigating these possible connections may reveal how tumor cells regulate different cell death programs under metabolic stress, and whether coordinated targeting of multiple RCDs could enhance therapeutic efficacy across diverse tumor contexts.
3. **Pathophysiological relevance**: Outside of cancer and immunity, does disulfidptosis contribute to tissue injury in neurodegeneration, ischemia-reperfusion, or inflammatory diseases where oxidative and metabolic stress are prominent?
4. **Metabolic plasticity**: How do cancer cells evade disulfidptosis, through rewiring NADPH production, modulating cystine uptake, or altering actin composition, and can these adaptations be therapeutically targeted?
5. **Immunological consequences**: Considering both immune-suppressive and immune-stimulating effects, what is the net outcome of disulfidptosis *in vivo*? Does it primarily impair immune effector cells, or can it also act as a form of ICD that enhances antitumor immunity? Resolving this paradox will be critical for therapeutic development.
6. **Biomarker development**: Can robust biomarkers be standardized to distinguish disulfidptosis from other RCDs? Without them, designing clinical trials or stratifying patients for disulfidptosis-inducing therapies will remain a major challenge.

While still in its infancy, the study of disulfidptosis holds significant potential. The next phase of research should focus on mechanistic dissection, biomarker development, and translational testing, ideally through integrated approaches that span biochemistry, cell biology, metabolism, immunology, and oncology. Studying this novel form of cell death pathway will not only reshape our understanding of redox-driven vulnerabilities but also open new therapeutic avenues for cancers and other diseases characterized by oxidative and metabolic stress.

## Figures and Tables

**Table 1.** Current pharmacological strategies for inducing or modulating disulfidptosis.

Strategy	Representative agents	Mechanism of action	Key outcome
**Glucose transport inhibition**	BAY-876, KL-11743 (GLUT inhibitors)	Block glucose uptake → reduce pentose phosphate pathway flux → NADPH depletion → impaired reduction of cystine to cysteine → accumulation of cystine-derived disulfides	Induces disulfide accumulation and cytoskeletal collapse under conditions with impaired glucose import
**Redox system inhibition**	Auranofin, Gaudichaudione H	Inhibit TXNRD1 and disrupt thiol–disulfide homeostasis	Amplifies disulfide stress and triggers disulfidptotic cell death
**Combination strategies**	GLUT inhibitors + ER stress modulators (e.g., GSK2656157, GSK2606414, Salubrinal) or other oxidative stress inducers	Dual suppression of reducing capacity and increased ROS burden	Synergistic induction of disulfidptosis in metabolically active tumors
**Nanomaterial-based approaches**	Cu-, Fe-, or Pd-based nanocatalysts	Catalytic oxidation of thiols and depletion of intracellular reducing agents	Enable spatial control and tumor-targeted induction of disulfidptosis

GLUT: glucose transporter; NADPH: reduced nicotinamide adenine dinucleotide phosphate; TXNRD1: Thioredoxin reductase 1; ER: endoplasmic reticulum; GSK: GlaxoSmithKline; ROS: rough mitochondrial reactive oxygen species.

## Data Availability

Not applicable.
